# Performance of Two Different Flight Configurations for Drone-Borne Magnetic Data

**DOI:** 10.3390/s21175736

**Published:** 2021-08-26

**Authors:** Filippo Accomando, Andrea Vitale, Antonello Bonfante, Maurizio Buonanno, Giovanni Florio

**Affiliations:** 1 Department of Earth, Environmental and Resources Sciences, University of Naples “Federico II”, 80126 Naples, Italy; andrea.vitale@unina.it (A.V.); gflorio@unina.it (G.F.); 2 Institute for Mediterranean Agricultural and Forest Systems (ISAFOM), Consiglio Nazionale delle Ricerche (CNR), 80055 Portici, Italy; antonello.bonfante@cnr.it (A.B.); maurizio.buonanno@cnr.it (M.B.)

**Keywords:** unmanned aerial vehicle, magnetic surveys, spectral analysis, magnetic noise, low-pass filtering

## Abstract

The compensation of magnetic and electromagnetic interference generated by drones is one of the main problems related to drone-borne magnetometry. The simplest solution is to suspend the magnetometer at a certain distance from the drone. However, this choice may compromise the flight stability or introduce periodic data variations generated by the oscillations of the magnetometer. We studied this problem by conducting two drone-borne magnetic surveys using a prototype system based on a cesium-vapor magnetometer with a 1000 Hz sampling frequency. First, the magnetometer was fixed to the drone landing-sled (at 0.5 m from the rotors), and then it was suspended 3 m below the drone. These two configurations illustrate endmembers of the possible solutions, favoring the stability of the system during flight or the minimization of the mobile platform noise. Drone-generated noise was filtered according to a CWT analysis, and both the spectral characteristics and the modelled source parameters resulted analogously to that of a ground magnetic dataset in the same area, which were here taken as a control dataset. This study demonstrates that careful processing can return high quality drone-borne data using both flight configurations. The optimal flight solution can be chosen depending on the survey target and flight conditions.

## 1. Introduction

Magnetic survey represents a rapid, non-invasive, and cheap prospection strategy, which makes it widely used in applied geophysics. The most common applications concern targets of geological, archaeological, and engineering interest. In recent years, unmanned aerial vehicles (UAVs), or drones, have become standard tools for acquiring spatial data to support various geoscientific analyses, reducing the cost and the time necessary to complete a survey, allowing extension of the prospections even over places of difficult access, and limiting the risks for field personnel. Several geophysical instruments have been mounted on UAVs as payloads (hyperspectral cameras, magnetometers, radar antennas, gamma-ray detectors), opening up the possibility of doing “drone geophysics”. A typical drone-borne geophysical survey has intermediate characteristics between the classical ground and airborne surveys in terms of spatial resolution. In the magnetic case, UAV and magnetometers were integrated in a variety of systems thanks to the development of miniaturized, lightweight magnetic sensors and acquisition boards [1,2,3,4,5,6,7,8,9,10,11]. The integration of UAV and magnetometers is opening new perspectives to the search and modelling of shallow magnetized sources. The possible fields of applications include topics of primary interest today, such as the field of mining exploration, the identification of active faults, or other geological targets. Drone magnetometry also has its role in engineering applications, such as archaeogeophysics and the preliminary studies to the decommissioning of oil industry infrastructures.

The acquisition of magnetic data from a plane, helicopter, or drone suffers from static and dynamic magnetic interferences from the platform, so that a number of compensation techniques were proposed aimed at minimizing these disturbances (see [12] for a list of studies dealing with these techniques, since 1950 to 2017). The traditional aeromagnetic compensation scheme is based on the attitude information, but it is not generally applied in the context of UAV-borne magnetic data. It would require performing some test flights in an area of nearly constant external magnetic field, a condition achievable at a high altitude [13]. In the drone-borne case, the problem is generally tackled by suspending the magnetometer sensors away from the UAV or by attempting some post-acquisition filtering of the data [14].

Several studies [4,6,7,8,10] have tested various magnetometer–UAV distances concluding that an optimal attenuation of the electromagnetic noise is obtained when the magnetometer sensor is 3–5 m away from the drone motors. In fact, this distance may vary according to the type of drone and payload. Thus, a solution generally accepted is to hang the magnetometer sensor to the drone with ropes, up to the distance of 3 m. However, this configuration of the UAV-magnetometer system could compromise the stability of the flight (e.g., in case of windy weather) and can create unwanted periodic variations in the acquired magnetic data generated by the inevitable oscillations of the magnetometer sensor. [9,11] quantify the impact of yaw, pitch, and roll on magnetic data acquired with a sensor suspended 3 m below the UAV.

This work focused on the comparison of the magnetic data acquired over near-surface targets (metallic pipes) using two different UAV-magnetometer arrangements. These strategies involved not only the use of different distances between the UAV and the magnetometer sensors but also the way the magnetometer is attached to the drone. We then checked the quality of the data acquired with the two configurations by comparing them to a magnetic ground data acquired in the same site. First, the characterization of the noise in the drone-borne data were assessed by a spectral analysis, then the comparison of the source depth estimates from the drone-borne and ground datasets helped us judge the value of the different data acquisition and post-processing strategies tested.

## 2. Measurements and Survey Design

The drone-borne magnetic and the ground magnetic surveys were conducted on 1 October 2020 at Verteglia Plain, a small tectonic-karst basin, located in the Picentini Mts. (southern Apennines, Italy). The Terminio-Tuoro massif is characterized by a carbonate succession in platform facies, with Jurassic and Cretaceous limestones covered by quaternary deposits, represented by discordant accumulations of pyroclastic products of Somma–Vesuvius activity and lacustrine deposits.

The surveys were performed during the “SEG Geophysical Field Camp in Southern Italy 2020” (for more information, see https://seg.org/fieldcamps accessed on 22 August 2021.) activities, organized by the SEG Student Chapter of the University of Naples Federico II and partially funded by the SEG Foundation. The investigated area is rather flat and devoid of dense vegetation.

The objective of the geophysical investigations in this area was to locate metal objects near the surface, such as steel pipes and cables, whose presence had been ascertained in the area by previous excavations. However, the precise location, shape, and number of sources were unknown.

### Drone-Borne and Ground Magnetic Surveys’ Design

**Figure 1:**
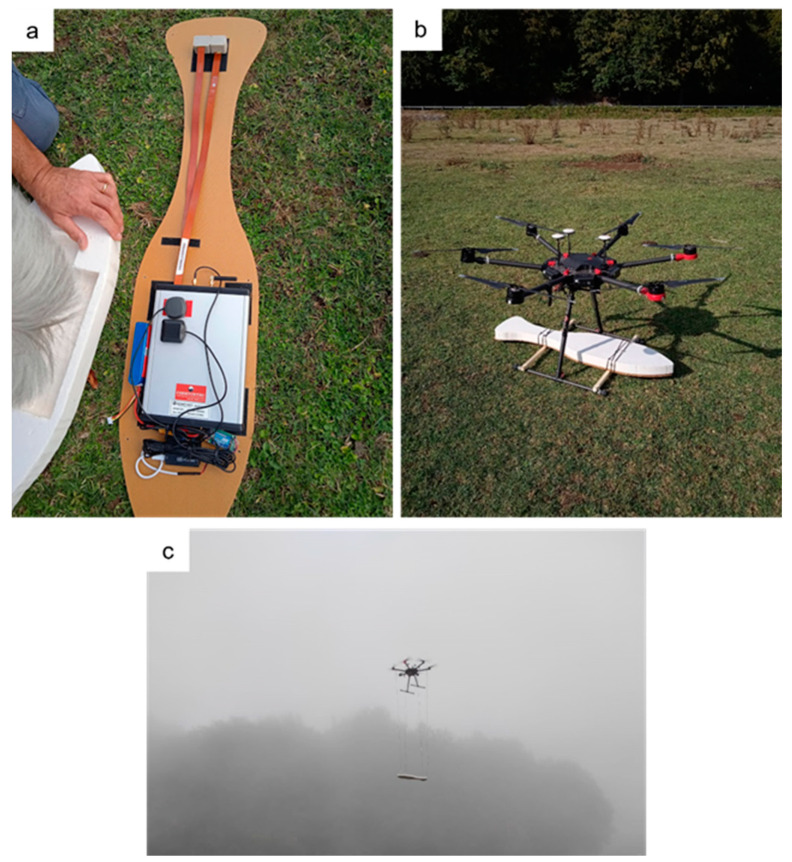
(**a**) Arrangement of the GEOMETRICS MFAM “Development kit” magnetometer inside the bird; (**b**) first flight configuration (F0.5), with the magnetometer fixed to the UAV landing sled, 0.5 m below the platform; (**c**) second flight configuration (F3.0), with the magnetometer suspended 3 m below the UAV.

For both aerial and ground surveys, we used a Geometrics Micro-Fabricated Atomic Magnetometer (MFAM) in the “Development kit” version, a laser-pumped atomic magnetometer with two alkali-vapor caesium sensors. The very high sampling rate of this magnetometer (1000 Hz) allows obtaining a unaliased measurement of 50 Hz fields as well as the correct identification of the high-frequency magnetic noise caused by the rotary aircraft used. In our prototype configuration, the MFAM is housed in a light, aerodynamic, and nonmagnetic custom bird made of polystyrene with a thin and rigid base made of a Nomex honeycomb sandwich panel (Figure 1a) (designed and assembled by the CNR-ISAFOM team). A GNSS receiver is included in the bird and records the position information at a 1 Hz rate during the magnetic data acquisition. The platform used for aerial survey was an electric-powered DJI Matrice M600 pro hexacopter. The total payload weight was about 2.0 kg. During the first flight configuration, the magnetometer was fixed to the UAV landing sled, with the MFAM sensors at a distance from the rotors of only 0.5 m (Figure 1b). During the second flight configuration, the MFAM was suspended 3 m below the hexacopter rotors by using thin nylon ropes (Figure 1c). For simplicity, from now on the flight configurations and the datasets relative to a magnetometer–UAV distance of 0.5 m and 3 m will be referred to as F0.5 and F3.0, respectively. To maximize the magnetic data quality and avoid falling in the sensor’s dead zone, in each survey the lines were oriented along a direction approximately North–South.

The two pre-programmed flights were conducted on the same 105 m × 24 m area, at a speed of 2 m/s, and at different elevations such as the magnetometer sensors being at about 8 m above ground level (AGL) in both flight configurations. Seven parallel survey lines oriented in the North–South direction were flown with an approximated 4 m line’s separation. With MFAM operating at 1000 Hz and a flight speed of about 2 m/s, the sampling step of the magnetic data along the survey lines was as small as about 2 mm. The geomagnetic field in this area had a declination of 4° and an inclination of 57°.

**Figure 2:**
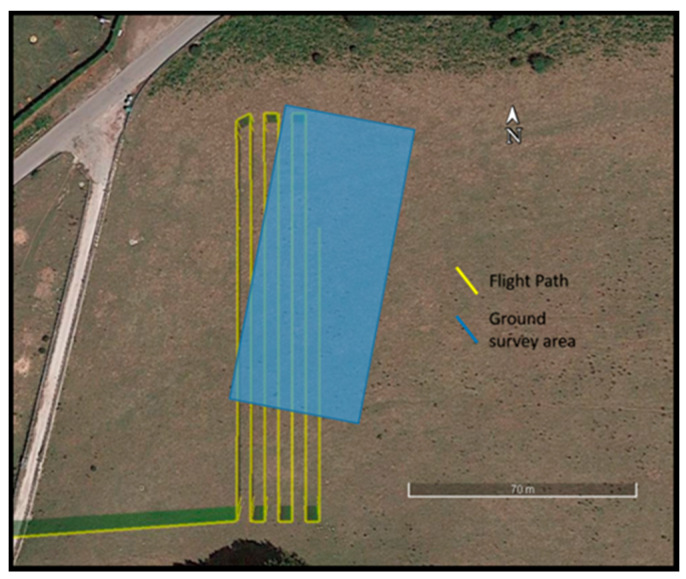
Planning of ground and aerial surveys.

The ground survey was conducted in a 90 m × 38 m area, partially overlapping with that covered by UAV surveys, along bidirectional lines spaced 2 m apart, oriented in a North–Northeast to South–Southwest direction (Figure 2). To minimize heading errors, the magnetometer was not rotated when starting a new line. The ground magnetic data were positioned with respect to a local reference system by introducing markers in the data stream at pre-defined positions along the profile (in our case, at the ends and at the center of each survey line).

The magnetic ground survey lasted approximately 2 h and was conducted by the participants to the SEG Geophysical Field Camp under the supervision of expert tutors; each of the two flights lasted approximately 10 min. During the time interval in which the drone-borne ground magnetic surveys were completed, the total magnetic field was monitored at a point in the same survey area. The total field variations were negligible, so that no temporal correction was applied to the three datasets.

## 3. Results

Here, we present the main features of the acquired data, which will be analyzed in detail in the Discussion section.

### 3.1. Drone-Borne Surveys

**Figure 3:**
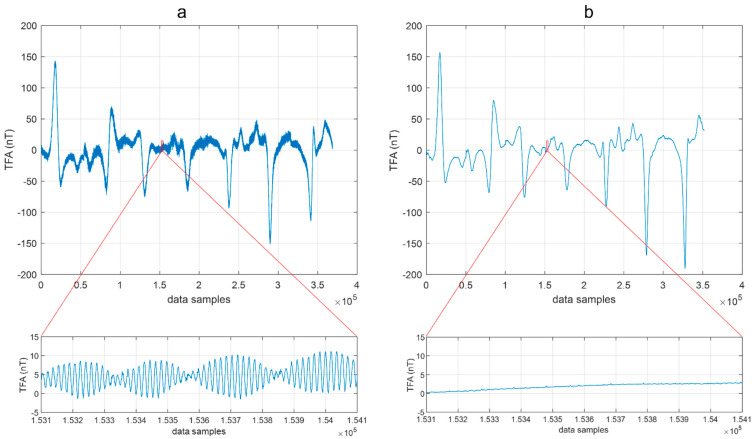
Total-field anomaly acquired using different flight configurations. (**a**) Data acquired with a magnetometer–UAV distance of 0.5 m (F0.5 dataset); (**b**) data acquired with a magnetometer–UAV distance of 3 m (F3.0 dataset). “TFA” stands for “total-field anomaly.”

The analysis of the data obtained by the MFAM magnetometer during flights included at its very first step the removal of all the values relative to the UAV turns between the survey lines and the subtraction of the IGRF. The resulting unfiltered total-field anomaly data of the two flights collected along the seven profiles are displayed in Figure 3. Here the data are shown sequentially, without using the position information. The difference between the data recorded in the two flight configurations is evident. Although the two datasets look very similar in the anomaly shapes and amplitudes, when the magnetometer was very near to the drone (F0.5; Figure 3a) a high-frequency and high-amplitude noise (maximum amplitude of about 10 nT) was present during all the flight duration.

Thus, we can reasonably associate this disturbance to the magnetic and electromagnetic interference fields generated by the UAV platform. As [15] showed, the UAV presents sources of magnetic and electromagnetic interference signals, with the last proportional to the rotation frequency of the electromagnetic motor. However, at an offset distance of 3 m, as in the F3.0 configuration, the magnetic field produced by a DJI M600 hexacopter appears sufficiently attenuated (Figure 3b), which is consistent with the results of the already mentioned previous studies about the optimal magnetometer–UAV distance.

**Figure 4:**
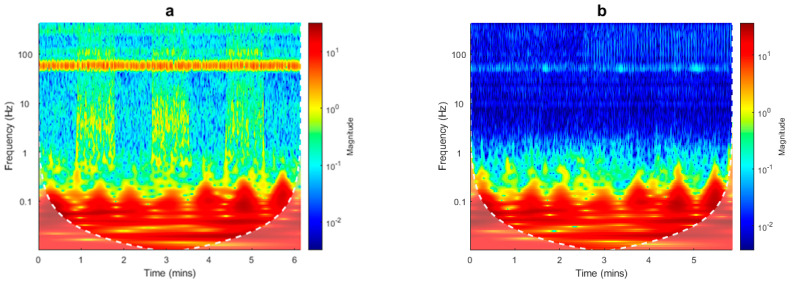
Spectral analysis of UAV data. (**a**) Scalogram of F0.5 dataset; (**b**) scalogram of F3.0 dataset. Edge effects in the computation of the spectra may be significant in the dimmed area below the dashed white line.

A spectral analysis of data acquired during both flight configurations can provide insights into the differences in the signal components in the two cases. The analysis of aeromagnetic data with wavelets is often an advantageous approach, due to the ability to explore the variations of the frequency content of the data through time or space. The discrete wavelet transform can be used to perform a localized filtering of the data [16], while in our case we used the continuous wavelet transform (CWT) to obtain a scalogram of the datasets in the time domain. This analysis helped us to identify the noise and signal components of the data. We used a “morse” wavelet, and we present the modulus of the complex set of CWT coefficients for the drone-borne datasets in Figure 4.

First, we noticed that the scalogram relative to the F0.5 configuration (Figure 4a) highlights, between 0.2 and 100 Hz, alternating amplitudes related to different survey lines. These different amplitudes can be associated with a strong heading error: the data acquired from South to North have a lower average value and noise amplitude than those acquired from North to South. The heading error is an intrinsic instrumental characteristic due to the orientation of the sensors. Its amplitude depends on the fact that the sensors are placed in the volume with the strong magnetic effect of the UAV. In fact, the UAV has its own magnetic effect, and the position of the magnetometer sensors with respect to this UAV-related magnetic effect may change with the flight direction, altering the average value and the noise amplitude of the recorded data from line to line. During the first flight, the sensors were very near to the UAV so that the heading error results were strongly amplified with respect to the F3.0 configuration, when the sensors were at 3 m from the UAV rotors.

**Figure 5:**
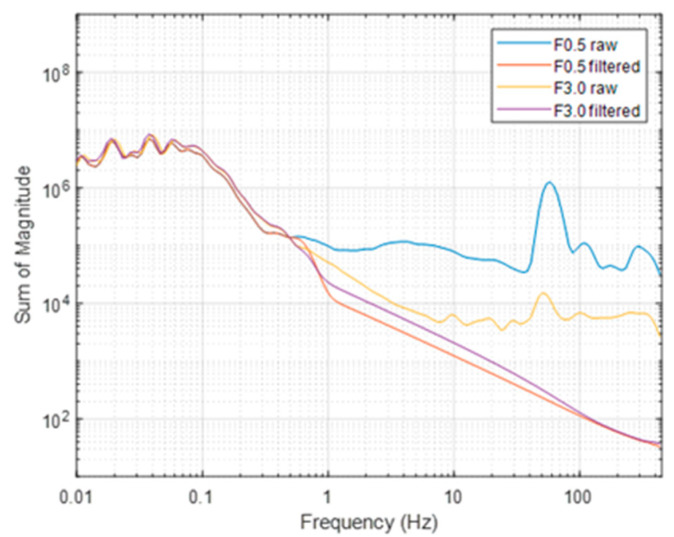
Sum of power of the scalograms for each frequency of both raw and filtered data flights.

For frequencies above 0.5 Hz, the F0.5 signal always has a higher magnitude than that of F3.0, with an evident peak around 50 Hz. This feature, highlighted by the scalogram analysis, can be interpreted by the presence of a significant platform-induced magnetic and electromagnetic interference sensed by the magnetometer. Ref. [15] shows that most of the electromagnetic interference caused by the drone engines has a main power spectral density peak at about 50 Hz. The data acquired with the F3.0 configuration was clearly less affected by magnetic interference from the UAV. However, the scalogram shows again a peak in the power spectral density around 50 Hz and, with much lesser amplitude, at 10, 20, and 30 Hz. To compare easily the two datasets for each flight, we computed the sum of the scalogram magnitudes at each frequency (Figure 5). It resulted that the scalograms of the two datasets were very similar for frequencies lower than 0.5 Hz.

To estimate, in the time-frequency scalograms (Figure 4), the frequency band associated to the target signal, similarly to what was done by [15], we can evaluate the ratio of the UAV speed (2 m/s) and the anomaly horizontal extent (representing a half-wavelength of the signal) multiplied by two. The analysis of the data profiles shows an anomaly half-wavelength of 15–25 m, so that we can estimate a target signal frequency band between 0.06 and 0.04 Hz. In fact, we can see that these are the frequencies where most of the power of the scalograms is concentrated (Figure 4).

**Figure 6:**
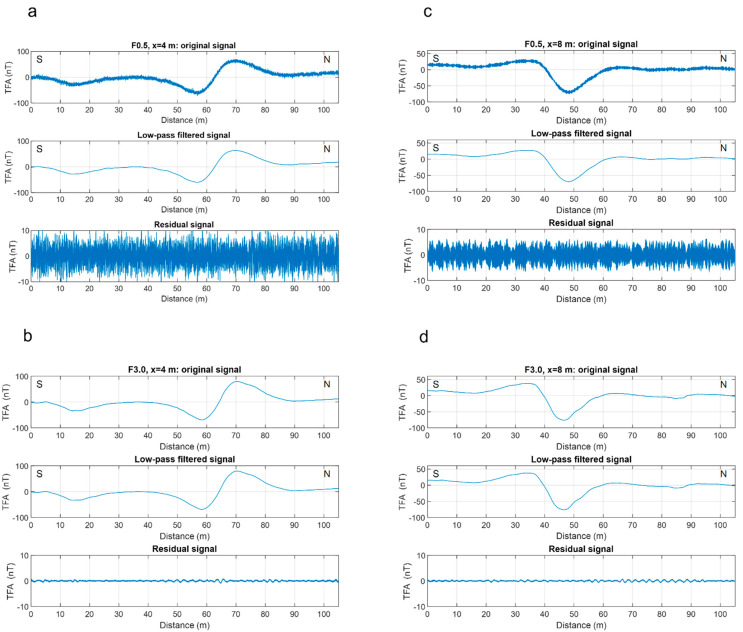
Application of a 0.5 Hz Hanning-window low-pass filter along two profiles for F0.5 and F3.0 datasets. The three plots in each panel show the raw data, the filtered data, and their difference (the residual signal). (**a**) Profile at x = 4 m; the magnetometer–UAV distance was 0.5 m; (**b**) profile at x = 4 m; the magnetometer–UAV distance was 3 m; (**c**) profile at x = 8 m; the magnetometer–UAV distance was 0.5 m; (**d**) profile at x = 8 m; the magnetometer–UAV distance was 3 m. The difference in amplitude between the residual signal in (**a**,**c**) demonstrates the heading error, as the two profiles were acquired while flying in opposite directions. “TFA” stands for “total-field anomaly.”

The above analysis guided us in the design of a time-domain Hanning-window low-pass filter with a cut-off frequency of 0.5 Hz, aimed at removing the noise and preserving the lowest frequency signals, associated with buried sources. This filter was applied to both the drone-borne datasets (Figure 5), as the overall characteristics of the scalogram are very similar in the two cases, differing mainly in the power of the frequencies higher than about 0.5 Hz. The results of application of such a filter to the raw magnetic data is clearly shown in Figure 5. An example of application of the low-pass filter along two profiles of both UAV datasets is presented in Figure 6.

**Figure 7:**
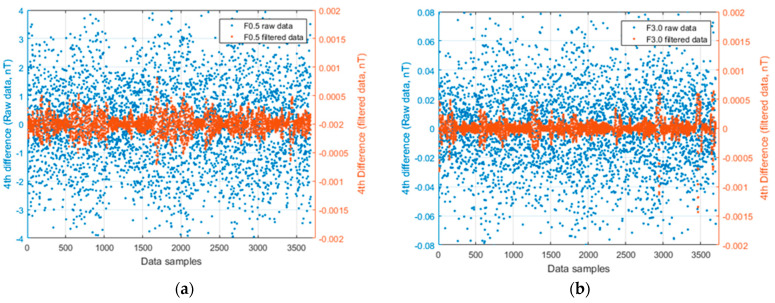
The calculated fourth difference of F0.5 (**a**) and F3.0 (**b**) datasets. Blue points refer to the raw data (amplitudes on the left *y*-axis), and red points refer to the filtered data (amplitudes on the right *y*-axis).

A commonly used method to evaluate the high-frequency noise level in aeromagnetic data is the computation of the amplitude of the “fourth difference,” a numerical approximation to the fourth derivative of measured data [17,18]. At the point where a central value ΔT_0_ is measured, the fourth difference (FDIFF) is evaluated as:

FDIFF = −(Δ*T*_−2_ − 4Δ*T*_−1_ + 6Δ*T*_0_ − 4Δ*T*_+1_ + Δ*T*_+2_)/16 (1)

where Δ*T*_−2_, Δ*T*_−1_, Δ*T*_+1_, and Δ*T*_+2_ are the four values measured at locations symmetric with respect to the central value. Generally, the accepted level for the noise envelope, as evaluated by the fourth difference, should be less than ±0.05 nT for data sampled at 10 Hz [19]. The F0.5 and F3.0 datasets were resampled at 10 Hz, and the fourth difference was evaluated (Figure 7). The unfiltered F0.5 data shows a strong noise, with an FDIFF ranging from −4 nT to 4 nT. However, after the filtering, the fourth difference values were well inside the ± 0.05 nT range. In the case of the F3.0 dataset, the unfiltered data were practically already inside the ± 0.05 nT range.

**Figure 8:**
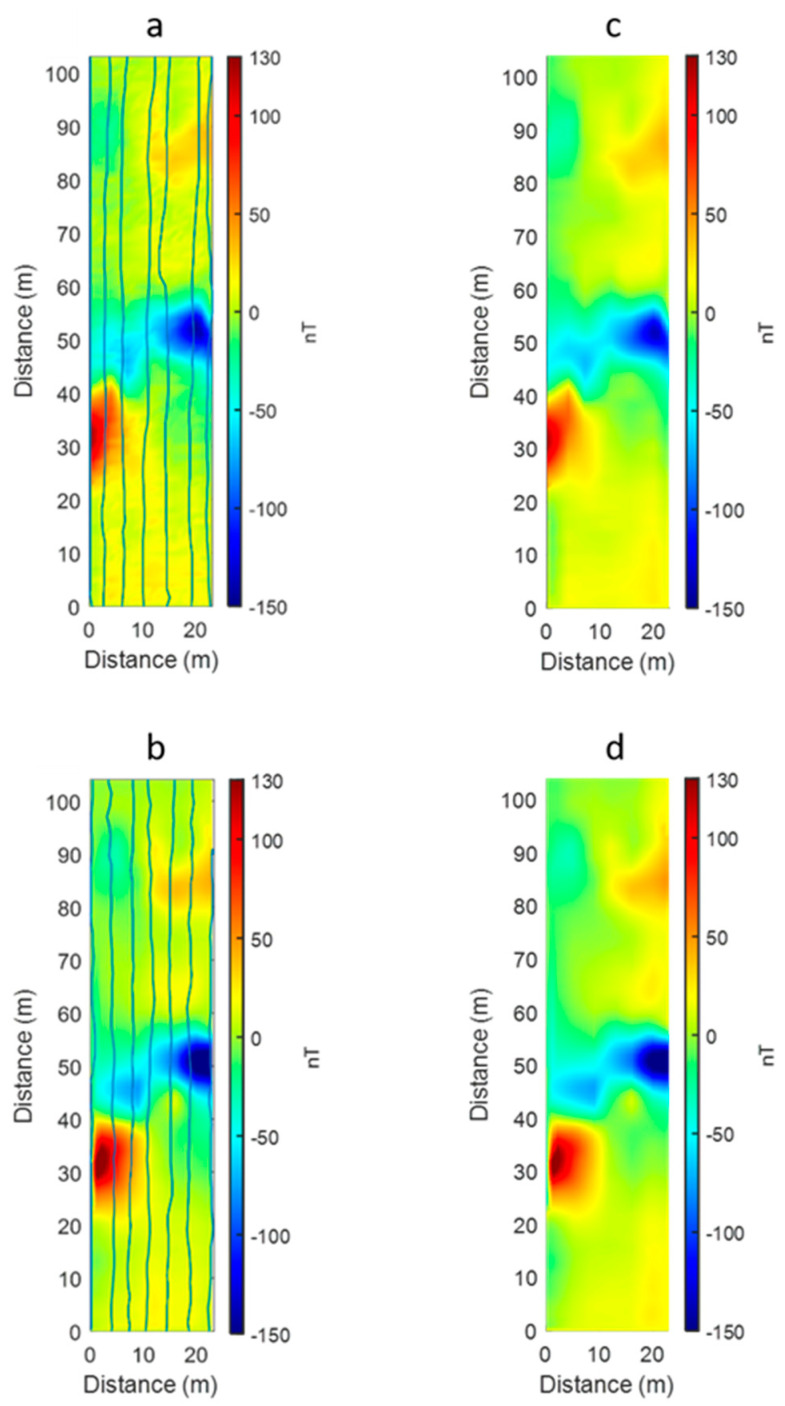
Magnetic anomaly maps. (**a**) F0.5 survey, raw data; (**b**) F0.5 survey, filtered data; (**c**) F3.0 survey, raw data; (**d**) F3.0 survey, filtered data. The blue lines in (**a**,**c**) represent the true flight paths during the two surveys.

As a standard processing step, we interpolated the data recorded along the seven profiles for the two drone-borne datasets to have a map of the magnetic anomalies in the area, using a gridding cell of 1 × 1 m^2^. The position, amplitude, and shape of the anomalies shown in the maps of the raw (Figure 8a,b) and filtered data (Figure 8c,d) were similar. The two total field maps showed a maximum amplitude variation of about 280 nT, ranging from about −150 to 130 nT, and we identified two main groups of anomalies, one trending NE–SW and located in the central sector, displaying the greatest amplitudes, and the other in the northern sector approximately trending in an E–W direction. Small amplitude differences between the two maps can be attributed to the different noise levels, to the inaccuracy of the flight altitude, which was set by GPS and which may have slightly differed between the two surveys, and to the inaccuracy of the GPS horizontal positioning in areas with strong magnetic gradients.

Based on previous information and on the magnetic anomalies’ shapes, we can infer that the sources of the anomalies were probably buried pipes and, perhaps, cables. In fact, the magnetic anomalies display the typical alternating magnetic highs and lows that can be measured above a metal pipe [20]. The individual sections of metal pipes acquire a permanent magnetization during manufacture, forming single magnetic dipoles. The direction of the magnetization varies from one pipe section to another depending on the orientation of the pipe, and this reflects in the systematic variation of the anomaly amplitudes, and/or sign, above a pipeline [20].

**Figure 9:**
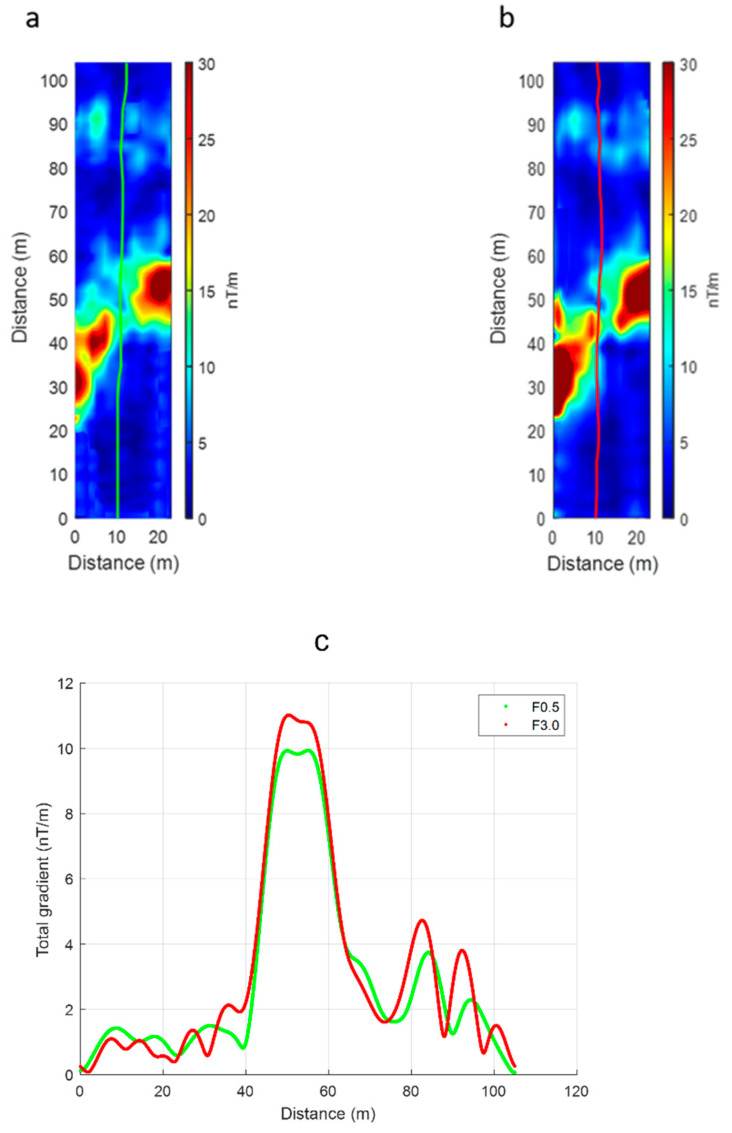
Total gradient maps relative to filtered F0.5 (**a**) and F3.0 (**a**) UAV datasets; (**c**) total gradient computed along a filtered profile (located by green and red lines in (**a**,**b**)). The green line refers to the F0.5 dataset, and the red line refers to the F3.0 dataset.

To better locate the buried sources of the magnetic anomalies in the area, we computed the total gradient, also known as the amplitude of the analytic signal [21]. In the total gradient maps (Figure 9), the anomaly sources should be located in correspondence to the maxima, except for possible small offsets that theoretically should not be present when computing the total gradient due to 2D sources along a data profile. The maps of Figure 9 clearly show the source position in the central sector, while in the northern zone the number and location of the sources appears more uncertain. The analysis of the total gradient computed along the data profiles (Figure 9c) can help to better distinguish all the sources present, because of the very high spatial detail (the sampling step is only 2 mm). From the analysis of all the data profiles the probable presence of linear anomalies elongated in the NE–SW direction can be recognized, with two of them in the northern area (80 m < y < 95 m) and one in the central area (20 m < y < 60 m). The intense anomaly in the central area may also be the result of the coalescence of the magnetic effects of two nearby linear structures.

### 3.2. Ground Survey Magnetic Data

**Figure 10:**
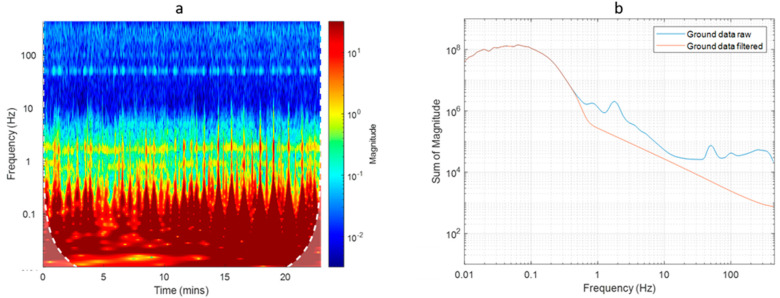
Spectral analysis of ground data. (**a**) Scalogram of ground data. Edge effects in the computation of the spectrum may be significant in the dimmed area below the dashed white line; (**b**) sum of power of the scalogram for each frequency.

The magnetic data of the ground survey were analyzed with the same procedures used for the UAV data. Interestingly, the scalogram of the ground data (Figure 10) appeared very similar to the scalogram of the F3.0 drone-borne dataset, relative to a magnetometer–UAV distance of 3 m (Figure 4b). In the ground-data scalogram, the frequency band relative to the target signal results was similar to that of the drone-borne data (compare Figure 4 and Figure 10) because the speed was then only about 1 m/s, while the half-wavelength of the anomalies of interest was reduced to about 10 m, implying again a frequency of about 0.05 Hz. In the ground data case, the peak at 50 Hz can be ascribed entirely to the power electrical lines present in the area. However, the same 50 Hz peak was also visible in the UAV data, with a magnitude very similar when the distance between the magnetometer and UAV rotors was set to 3 m. This similarity between the 50 Hz peak amplitude between ground data and F3.0 data suggests that magnetic data are relatively free of UAV-generated noise when the magnetometer sensors are at a distance of at least 3 m from the UAV. Other minor peaks appeared at different frequencies in the ground and UAV scalograms. These peaks cannot be related to oscillations of the magnetometer during the data acquisitions, as the spectral content of the attitude data (not shown) showed peaks at higher frequencies, associated to a very low power.

As the power of the target signal was concentrated at frequencies similar than in the UAV case, to remove the power related to the noise and preserve the useful signal at lower frequencies, we applied to the ground data along each profile the same low-pass filter designed for the UAV data (cut-off frequency of 0.5 Hz, using a Hanning window). The result of the filtering and the comparison with the original data is presented in Figure 10b as the sum of power of the scalogram for each frequency.

**Figure 11:**
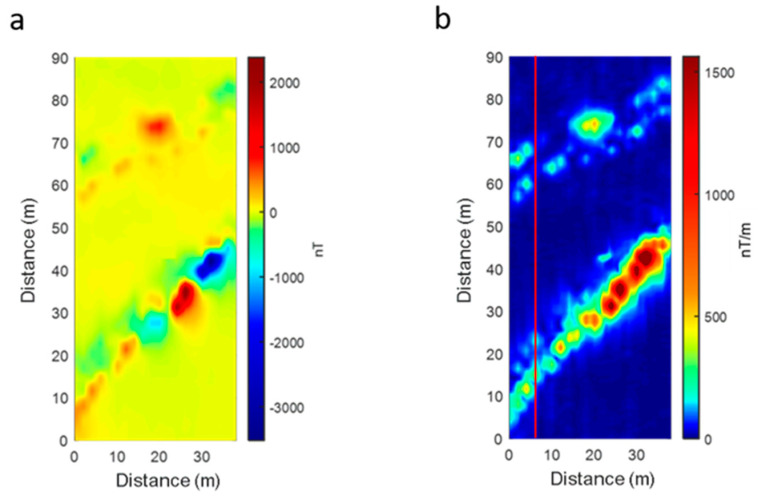

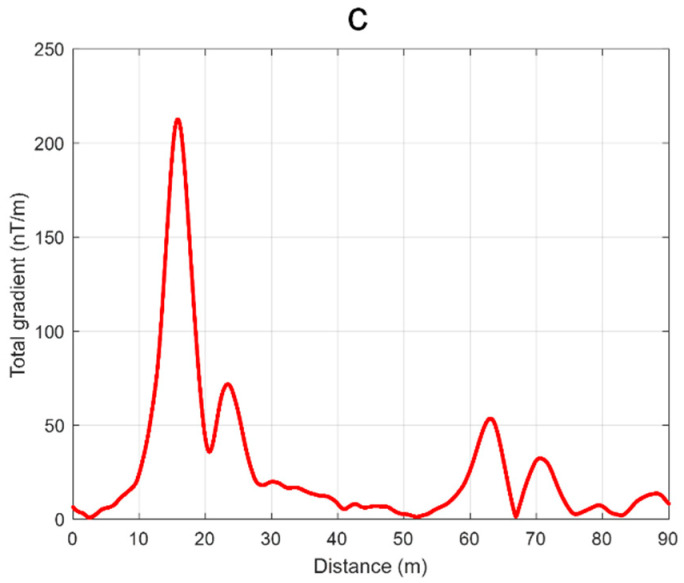
Ground survey dataset. (**a**) Total field anomaly map; (**b**) total gradient map; (**c**) total gradient of the profile data at x = 6 m (red line in Figure 11b). The secondary total gradient peak (at x = 25 m in this data profile) is sometimes visible on other profiles and could be related to another linear source.

The ground total-field anomaly data were interpolated on the survey area using a gridding cell of 0.5 × 0.5 m^2^, and the total gradient was also computed to allow for an easier horizontal location of the sources (Figure 11). The main features of the magnetic anomaly map are the presence of the typical alternating magnetic highs and lows that can be measured above a metal pipe [20] and already described for the UAV data (Figure 8). Here the magnetic anomalies are sampled at a higher resolution and clearly show the presence of two NE–SW linear sources in the northern area (55 m< y< 85 m), about 10 m apart from each other, and probably just one linear source in the central area (0 m < y < 50 m), although the presence of another source located north of the main one cannot be completely excluded (Figure 11b,c).

### 3.3. Source Depth Estimation

The estimate of the source depth from drone-borne data can represent a quantitative quality control when compared to depths obtained from ground data, which we can consider as a reference for clean and valid data. With this modelling, we wanted to check if the different conditions in which the magnetic field was measured during the flights with the magnetometer at 0.5 m and 3 m from the drone and the successive data filtering might have distorted the signal or, conversely, if the integrity of the target signal was preserved [22].

**Figure 12:**
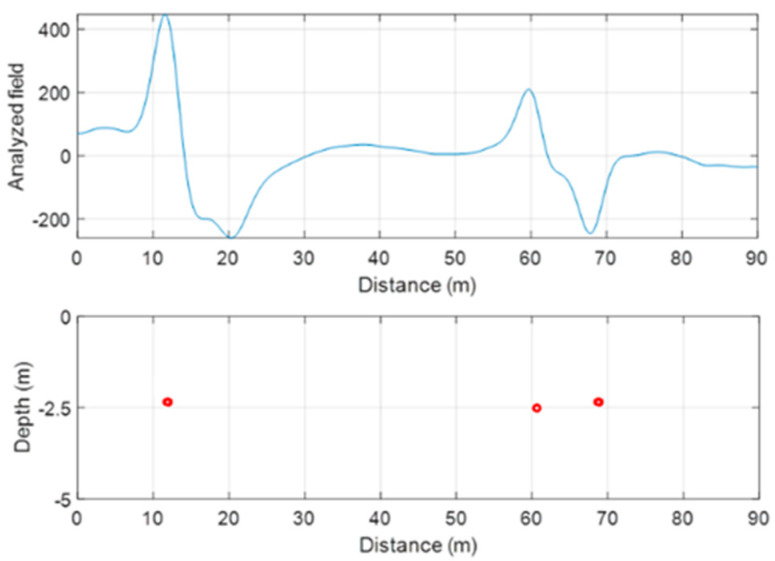
Example of the Euler deconvolution application to profile data. (**a**) Ground survey total field profile at x = 4 m; (**b**) Euler deconvolution solutions (red dots), localized at the position of the center of the sources of the magnetic anomalies.

Euler deconvolution is a semi-automated method to estimate the source depth from potential field data, which is based on the Euler homogeneity of the magnetic field generated by simple sources [23,24]. In its standard version, the method assumes an integer “structural index” value, associated with simple sources (dipole, line, sheet, half-space). In the case in study, consistently with the presence of linear sources (Figure 8 and Figure 11), we could safely assume a structural index value equal to 2, characterizing a horizontal line of dipoles. We applied this technique to the profile data acquired during both UAV and ground surveys, as they were characterized by a very small spatial sampling step (Figure 12). This should imply an advantage in terms of results accuracy with respect to the analysis of the maps, as they are obtained by interpolation using a much larger grid step size. However, the surveyed profiles do not cross perpendicularly these two-dimensional sources, so that a correction factor was applied to the estimated depths, equal to the cosine of the angle between the profile direction and a line perpendicular to the source [25]. Consistently with the model of horizontal line of dipoles, the estimated depths can be considered as relative to the center of the buried pipes. The Euler deconvolution analyzes the data profiles with a moving-window approach, producing many solutions. In our case, the window size chosen was of 2.5 m (501 points). The selection of best solutions was based on the error associated to the depth estimate obtained by the least squares solution to the overdetermined linear system of equations [24].

As already pointed out, the number of sources clearly visible along the data profiles varied from UAV and ground datasets, probably because of coalescence effects at the flight altitude. Thus, we obtained two groups of Euler solutions for UAV data (S1 and S2, relative to anomalies located in the central and northern sectors, respectively) and three groups from ground data (L1 in the central sector and L2 and L3 in the northern one).

The estimated depths were significantly consistent for the three different datasets, so that the results are very satisfying (Table 1 and Table 2). In detail, the data acquired in the F0.5 configuration provided average depths of 1.76 m for the source of anomaly S1, which was 7% less that the average depth (1.9 m) obtained from the F3.0 configuration and ground datasets. In the northern sector, UAV data individuate a single anomaly, and in this case the depth estimated from two aerial datasets were consistent (average depth of 1.76 m). On the other hand, the ground survey had a sufficient resolution to separate the effects of two linear sources at average depths of 1.7 and 1.8 m, showing a very good agreement with the depth estimated from drone-borne magnetic data.

## 4. Discussion

In this study, we tested the quality of drone-borne magnetic data acquired using two different flight configurations. In the first configuration, the magnetometer was fixed to the landing sled at a distance of 0.5 m to the rotors, while in the second configuration the magnetometer was suspended 3 m below the UAV. The two configurations illustrate the possible solutions to obtain, on the one hand, the stability of the UAV-magnetometer system during flight and, on the other hand, the minimization of the magnetic and electromagnetic noise generated by the mobile platform. As was well shown by [10], the periodic swinging of the magnetometer during flight, when the magnetometer is suspended to the drone, may induce a noise at a characteristic frequency, which depends on the magnetometer–UAV distance, while the magnetic and electromagnetic noise related to the drone is concentrated at high frequencies, with a main peak at around 50 Hz. It is clear that the need to stabilize the magnetometer during flight and to minimize the UAV-related noise solicit the implementation of contrasting strategies, so that the solutions tested in this study can be considered as end-members of possible flight configurations for a magnetometer-UAV system.

We compared the power spectral densities of the datasets collected during the UAV flights and the ground survey. This comparison allowed us to distinguish the frequency contribution due to the target signal from that due to the magnetostatic and electromagnetic interference of the mobile platform. [15] carefully studied the magnetic and electromagnetic interference components generated by the DJI M600 hexacopter and as recorded by a Geometrics 1000 Hz MFAM sensor, which is the same drone-magnetometer system used in the present study. They distinguished four sources of interference signals, namely, (a) the mechanical rotation of the motors, (b) the permanent magnets in the motors, (c) the electrical commutation due to the passage of an AC current in the solenoids of the motor, and (d) the induced magnetic field generated by the ferromagnetic cores of the motor’s armatures. During flight, these interference components are characterized by a main peak frequency at 50 Hz, with part of the power centered at higher frequencies [15]. Some of these components may even be aliased in the 1000 Hz sampled magnetic field [15]. What was observed in the scalograms relative to both the drone-borne datasets (Figure 4) was consistent with these findings.

The free periodic swinging of a 3-m suspended magnetometer should induce an interference signal with a central peak at about 0.38 Hz [15]. The amplitude of this interference component was influenced by (a) the UAV speed and (b) the magnitude of the horizontal and vertical gradients of the magnetic field in the volume of the oscillations. This peak was not clearly visible in our data (Figure 5) because it had a relatively high power only in the data acquired during UAV turns between the survey lines, but these low-quality data were removed in a preliminary processing step. However, we processed the data relative to the turns of the drone between adjacent lines (including the first samples along each line) and could verify, by the computation of the CWT scalogram not shown, the presence of a magnitude peak at the expected frequency of 0.38 Hz, as predicted by [10] for a F3.0 flight configuration.

The main difference between the scalograms shown in Figure 4, relative to the F0.5 and the F3.0 configurations (UAV rotors–magnetometer sensors distance of 0.5 m and 3 m, respectively), was relative to frequencies higher than 0.5 Hz. In fact, when the magnetometer sensor was very near to the UAV, the power associated with frequencies higher than about 2 Hz increased to about one order of magnitude with respect to the F3.0 flight configuration. In the case studied in this article, the combination of the wavelength of the target signal and the survey speed assured a clear spectral separation between the primary target frequencies (<0.5 Hz) and the primary platform interference frequency (50 Hz) observed. This allowed a clean separation of these contributions using a low-pass filter with a cutoff frequency of 0.5 Hz. This cutoff frequency was higher than that used in other studies (e.g., [10] used a 0.2 Hz cutoff frequency), thus better preserving the integrity of the target signal. It is important here to highlight the role of the UAV speed on the time-domain spectral frequency of the target signal. In the case studied in this article, a speed of 5 m/s would have increased the target signal frequency to 0.125 Hz, implying a possible overlap with noise components.

Another difference between the F0.5 and F3.0 datasets regards the amplitude of the heading error, associated with opposite flight directions during the survey. To quantify the heading error from our dataset is not an easy task, as we did not acquire data along the same profile in opposite directions. However, we tried to obtain some insights by comparing the residual signal after filtering along adjacent profiles, as shown for two of them in Figure 6. The different amplitude of the removed signal was attributable to the different heading error when flying in opposite directions. We could estimate a static offset of +/−7 nT between opposite flight lines in the F0.5 configuration and of +/−0.15 nT between opposite flight lines in the F3.0 configuration. These values were related to the drone-magnetometer configurations used in this study and should be re-evaluated for other systems. However, they highlight the difference in amplitude of this effect for the two flight configurations used.

As already mentioned, the spectral content of ground data was very similar to that of the aerial dataset when the magnetometer was far from the drone, demonstrating that a distance of 3 m is sufficient to effectively attenuate most of the electromagnetic noise for this specific UAV-magnetometer system.

Finally, the very similar depth to source obtained from modelling the low-pass filtered drone-borne magnetic data and ground data demonstrate the soundness of the post-processing strategies.

## 5. Conclusions

In conclusion, our final recommendations are the following:

- the magnetometer could be placed even very near to the UAV in all the cases when it is necessary increasing flight security and stability (for example, in case of rugged terrain, in the presence of vegetation near the programmed flight altitude, or in case of non-optimal weather conditions);
- in general, the flight speed should be sufficiently slow to favor a good spectral separation between noise and signal, and this is especially important if the UAV–magnetometer distance is small;
- if positioned very near to the drone, a good practice should be to program the flight without performing the 180° turn at the end of each line, thereby avoiding strong heading errors—this procedure is simply implemented using flight programming software;
- the use of a magnetometer operating at a fast acquisition rate (in our case, 1000 Hz) is strongly advised to adequately sample the high frequencies (50 Hz or more) typically associated to UAV electromagnetic noise. This noise, if in aliasing, can contaminate the frequency band associated to the useful signal.

The results presented in our study regard the survey over intense magnetic anomalies, such as those generated by near-surface metallic objects. In case of weaker magnetic effects (e.g., in archaeological areas or for some mining applications), a very short UAV–magnetometer distance, similar to the first used in the present study, may not produce satisfying results, and further research is needed to assess the optimal flight configuration. In these cases, the amplitude of the target signal and that of the noise floor may be similar, and a simple filtering, as described in this article, might not be sufficient to obtain a clean signal.

## Figures and Tables

**Table 1:** Estimated depths along the profiles acquired with F0.5 and F3.0 configuration.

	F0.5 Configuratio		F3.0 Configuration	
	S1 Anomaly	S2 Anomaly	S1 Anomaly	S2 Anomaly
**Profile 1**	1.8 m	1.8 m	2.0 m	1.5 m
**Profile 2**	1.6 m	1.8 m	2.0 m	1.8 m
**Profile 3**	1.6 m	1.8 m	1.8 m	1.8 m
**Profile 4**	1.8 m	1.8 m	2.0 m	1.8 m
**Profile 5**	1.8 m	1.5 m	1.8 m	1.8 m
**Profile 6**	1.8 m	1.8 m	1.8 m	1.8 m
**Profile 7**	1.8 m	1.8 m	1.8 m	1.8 m
**Mean Value**	1.74 m	1.76 m	1.9 m	1.76 m
**Standard deviation**	0.10 m	0.11 m	0.11 m	0.11 m

**Table 2:** Estimated depths along the profiles of the ground survey.

		Ground Survey		
	L1 Anomaly	L2 Anomaly	L3 Anomaly
**Profile 1**	1.9 m	1.5 m	1.9 m
**Profile 2**	1.5 m	1.9 m	1.5 m
**Profile 3**	1.9 m	1.9 m	1.9 m
**Profile 4**	1.9 m	1.9 m	1.9 m
**Profile 5**	1.9 m	1.9 m	1.9 m
**Profile 6**	1.9 m	1.5 m	1.9 m
**Profile 7**	1.9 m	1.9 m	1.9 m
**Profile 8**	1.9 m		1.5 m
**Profile 9**	1.9 m		1.9 m
**Profile 10**	1.9 m	1.5 m	1.9 m
**Profile 11**	1.9 m		1.9 m
**Profile 12**	1.9 m	1.9 m	1.9 m
**Profile 13**	1.9 m	1.5 m	1.5 m
**Profile 14**	1.9 m	1.5 m	1.9 m
**Profile 15**	1.9 m		1.9 m
**Profile 16**	1.9 m	1.9 m	1.9 m
**Profile 17**	1.9 m	1.9 m	1.9 m
**Profile 18**	1.9 m	1.5 m	1.5 m
**Profile 19**	2.0 m	2.0 m	2.0 m
**Profile 20**	2.0 m	2.0 m	2.0 m
**Mean value**	1.89 m	1.7 m	1.81 m
**Standard deviation**	0.10 m	0.21 m	0.19 m

## Data Availability

The data that support the findings of this study are available from the corresponding author upon reasonable request.

## References

[1] Samson C., Straznicky P., Laliberté J., Caron R.M., Ferguson S., Archer R. (2010). Designing and building an unmanned aircraft system for aeromagnetic surveying. SEG Expanded Abstracts, Proceedings of the Society of Exploration Geophysicists 80th Annual International Meeting, Denver, CO, USA, 17–22 October 2010.

[2] Stoll J., Móritz D. (2013). Unmanned aircraft systems for rapid near surface geophysical measurements. International Archive of Photogrammetry, Remote Sensing and Spatial Information Science, Proceedings of the 75th EAGE Conference and Exhibition—Workshops, London UK, 10–13 June 2013.

[3] Cunningham M., Samson C., Wood A., Cook I., Doyle B. Detecting mineral ore bodies with UASs instrumented with magnetometers. Proceedings of the Unmanned Systems Canada Conference.

[4] Parvar K. (2016). Development and Evaluation of Unmanned Aerial Vehicle (UAV) Magnetometry Systems. Master’s Thesis.

[5] Wood A., Cook I., Doyle B., Cunningham M., Samson C. (2016). Experimental aeromagnetic survey using an unmanned air system. Lead. Edge.

[6] Cunningham M. (2018). Aeromagnetic Surveying with Unmanned Aircraft Systems. Master’s Thesis.

[7] Malehmir A., Dynesius L., Paulusson K., Paulusson A., Johansson H., Bastani M., Wedmark M., Marsden P. (2017). The potential of rotary-wing UAV-based magnetic surveys for mineral exploration: A case study from central Sweden. Lead. Edge.

[8] Parvar K., Braun A., Layton-Matthews D., Burns M. (2018). UAV magnetometry for chromite exploration in the Samail ophiolite sequence, Oman. J. Unmanned Veh. Syst..

[9] Walter C.A., Braun A., Fotopoulos G. (2019). Impact of 3-D attitude variations of a UAV magnetometry system on magnetic data quality. Geophys. Prospect..

[10] Walter C., Braun A., Fotopoulos G. Spectral Analysis of Magnetometer Swing in High-Resolution UAV-borne Aeromagnetic Surveys. Proceedings of the 2019 IEEE Systems and Technologies for Remote Sensing Applications Through Unmanned Aerial Systems (STRATUS).

[11] Walter C., Braun A., Fotopoulos G. (2020). High-resolution unmanned aerial vehicle aeromagnetic surveys for mineral exploration targets. Geophys. Prospect..

[12] Noriega G., Marszalkowski A. (2017). Adaptive techniques and other recent developments in aeromagnetic compensation. First Break..

[13] Tuck L., Samson C., Polowick C., Laliberté J. (2019). Real-time compensation of magnetic data acquired by a single-rotor unmanned aircraft system. Geophys. Prospect..

[14] Mu Y., Zhang X., Xie W., Zheng Y. (2020). Automatic detection of near-surface targets for unmanned aerial vehicle (UAV) magnetic Survey. Remote. Sens..

[15] Walter C., Braun A., Fotopoulos G. (2021). Characterizing electromagnetic interference signals for unmanned aerial vehicle geo-physical surveys. Geophysics.

[16] Paoletti V., Fedi M., Florio G., Rapolla A. (2007). Localized Cultural Denoising of High-Resolution Aeromagnetic Data. Geophys. Prospect..

[17] Hood P.J., Teskey D.J. (1989). Aeromagnetic gradiometer program of the Geological Survey of Canada. Geophysics.

[18] Teskey D.J., Barlow R., Hood P.J., Lefebvre D., Paterson N., Reford M., Watson D. (1991). Guide to aeromagnetic specifications and contracts. Geol. Surv. Can..

[19] Coyle M., Dumont R., Keating P., Kiss F., Miles W. (2014). Geological Survey of Canada aeromagnetic surveys: Design, quality assurance, and data dissemination. Geol. Surv. Can..

[20] Sowerbutts W.T.C. (1988). The use of geophysical methods to locate joints in underground metal pipelines. Q. J. Eng. Geol..

[21] Roest W.R., Verhoef J., Pilkington M. (1992). Magnetic interpretation using the 3-D analytic signal. Geophysics.

[22] Florio G., Fedi M., Pašteka R. (2014). On the estimation of the structural index from low-pass filtered magnetic data. Geophysics.

[23] Thompson D.T. (1982). EULDPH: A new technique for making computer-assisted depth estimates from magnetic data. Geophysics.

[24] Reid A.B., Allsop J.M., Granser H., Millett A.J., Somerton I.W. (1990). Magnetic interpretation in three dimensions using Euler deconvolution. Geophysics.

[25] Milsom J. (2003). Field Geophysics.

